# Deficiency of the LIM-Only Protein FHL2 Reduces Intestinal Tumorigenesis in *Apc* Mutant Mice

**DOI:** 10.1371/journal.pone.0010371

**Published:** 2010-04-28

**Authors:** Charlotte Labalette, Yann Nouët, Florence Levillayer, Sabine Colnot, Ju Chen, Valere Claude, Michel Huerre, Christine Perret, Marie-Annick Buendia, Yu Wei

**Affiliations:** 1 Département de Virologie, Institut Pasteur, Paris, France; 2 Inserm U579, Paris, France; 3 Département d'Endocrinologie Métabolisme et Cancer, Institut Cochin, Paris, France; 4 Inserm U567, Paris, France; 5 Department of Medicine, University of California San Diego, La Jolla, California, United States of America; 6 Département d'Anapathologie, Hôpital Bégin, Saint Mandé, France; 7 Département d'Infection et Epidémiologie, Institut Pasteur, Paris, France; The University of Hong Kong, Hong Kong

## Abstract

**Background:**

The four and a half LIM-only protein 2 (FHL2) is capable of shuttling between focal adhesion and nucleus where it signals through direct interaction with a number of proteins including β-catenin. Although FHL2 activation has been found in various human cancers, evidence of its functional contribution to carcinogenesis has been lacking.

**Methodology/Principal Findings:**

Here we have investigated the role of FHL2 in intestinal tumorigenesis in which activation of the Wnt pathway by mutations in the adenomatous polyposis coli gene (Apc) or in β-catenin constitutes the primary transforming event. In this murine model, introduction of a biallelic deletion of *FHL2* into mutant *Apc^Δ14/+^* mice substantially reduces the number of intestinal adenomas but not tumor growth, suggesting a role of FHL2 in the initial steps of tumorigenesis. In the lesions, Wnt signalling is not affected by FHL2 deficiency, remaining constitutively active. Nevertheless, loss of FHL2 activity is associated with increased epithelial cell migration in intestinal epithelium, which might allow to eliminate more efficiently deleterious cells and reduce the risk of tumorigenesis. This finding may provide a mechanistic basis for tumor suppression by FHL2 deficiency. In human colorectal carcinoma but not in low-grade dysplasia, we detected up-regulation and enhanced nuclear localization of FHL2, indicating the activation of FHL2 during the development of malignancy.

**Conclusions/Significance:**

Our data demonstrate that FHL2 represents a critical factor in intestinal tumorigenesis.

## Introduction

The four and a half LIM-only protein 2 (FHL2) contains only LIM domain (named after lin-11, mec-3 and Islet-1), which is a protein-protein interaction domain. FHL2 has recently emerged as a signalling protein that is critical in the transduction of signals from extracellular environment and in the control of gene expression program in response to different stimuli. In the cytoplasm, FHL2 interacts with α- and β-integrin subunits and focal adhesion kinase at focal adhesions where integrins bind extracellular matrix (ECM) [1]–[3]. FHL2 is involved in ECM-integrin receptor interaction, assembly of ECM proteins on the cell surface and bundling of focal adhesions [4], [5]. In response to external stimuli, FHL2 is translocated to the nucleus where it plays the role of transcription cofactor by interacting with numerous transcription factors and coregulators including androgen receptor (AR), AP1, CREB, PLZF, WT1, SKI, β-catenin, FOXO1, Runx2, serum response factor (SRF), E4F1 and CBP/p300 [6]–[18]. Mice deficient of FHL2 display defects in response to divers stimuli, including cardiac hypertrophy under β-adrenergic stimulation, healing defects in skin and intestinal wound and attenuated neovascularization after corneal injury [19]–[22].

Several lines of evidence suggest a role of FHL2 in carcinogenesis. *FHL2^−/−^* fibroblasts are temporarily resistant to oncogenic Ras-induced transformation [23]. FHL2 suppression inhibits anchorage-independent growth of cancer cell lines and tumor formation in immuno-compromised mice [24]. Up-regulation of FHL2 has been found in many human cancers [3], [13], [24]–[26]. Several studies have associated high level and nuclear expression of FHL2 with the aggressiveness of cancer and bad prognostic [6], [27] [28]. Moreover, numerous FHL2-interacting proteins including AR, AP1, PLZF, SKI, WT1, β-catenin, BRCA1 and E7 of human papillomavirus 16 [29], [30] have primary roles in various human cancers, implying that FHL2 may participate in transformation process through effects on the oncogenic activity of its partners.

We have previously demonstrated that FHL2 interacts with β-catenin and cooperates with CBP/p300 to stimulate transcriptional activity of the β-catenin/TCF4 complex [13], [18]. β-catenin is a key effector of the Wnt signalling pathway that has central roles not only in embryogenesis and tissue homeostasis but also in tumorigenesis (for review, see [31]). In human colorectal cancer, an overwhelming majority of cases carry mutations in Wnt pathway components including the adenomatous polyposis coli gene (Apc), axin and β-catenin. All the mutations lead to constitutive activation of the Wnt signalling pathway characterized by the formation of constitutive nuclear β-catenin/TCF complex. Genetic modelling of activation of the Wnt signalling pathway in the intestine has been made possible by the generation of *Apc* mutant mice that recapitulate human colonic carcinogenesis. These mice, epitomized by the *Apc^Min/+^* mouse, have been used intensively to test genetically the ability of candidate genes to enhance or repress adenoma formation *in vivo*.

In this study, we used the *Apc^Δ14/+^* model to address the contribution of FHL2 activity to the intestinal transformation process. *Apc^Δ14/+^* mice in which exon 14 of the *Apc* gene is deleted in one allele spontaneously develop multiple polyps along their intestinal tract [32]. All tumors lose the *Apc* wild type (wt) allele, accumulate β-catenin in the nucleus and overexpress the β-catenin target genes cyclin D1 and c-myc [32]. Using a genetic approach, we demonstrate that loss of FHL2 significantly suppresses tumor multiplicity in *Apc^Δ14/+^* mice. Our analysis of FHL2 expression in murine and human intestinal lesions revealed its progressive up-regulation and enhancement of nuclear expression during disease development. These results provide unequivocal evidence of the implication of FHL2 activity in the transformation process.

## Results

### FHL2 deficiency reduces intestinal polyposis in *Apc^Δ14/+^* mice

**Figure 1:**
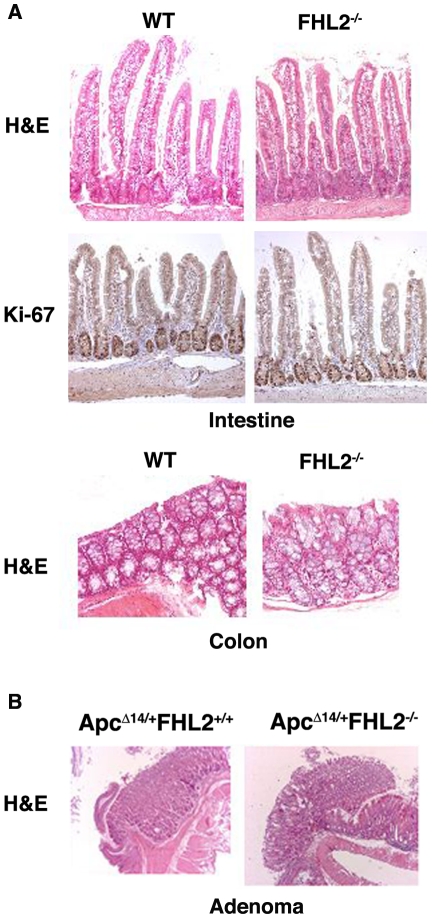
Histological analysis of normal intestine in *FHL2^−/−^* mice and intestinal adenomas in *Apc^Δ14/+^FHL2^−/−^* mice. A. Normal intestinal architecture in *FHL2^−/−^* mice. Intestine and colon sections from 11 month-old wt and *FHL2^−/−^* mice were stained with H&E or Ki-67 by immunostaining. B. Intestinal adenomas in *Apc^Δ14/+^FHL2^+/+^* and *Apc^Δ14/+^FHL2^−/−^* mice. Original magnifications, X100 (A and B).

To determine the *in vivo* effects of FHL2 in intestinal tumorigenesis, we took advantage of *FHL2^−/−^* and *Apc^Δ14/+^* mice to investigate how FHL2 affects the phenotypes associated with *Apc* loss [32], [33]. Despite defects in bone formation [15], [34], *FHL2^−/−^* mice live a normal lifespan [33]. Histological analysis of intestinal epithelium in *FHL2-null* mice revealed no major alterations in crypt-villus architecture of the small intestine and in the crypt structure of the colon (Fig. 1A). Moreover, *FHL2*-deficient cells remained in cycle in the crypt, as demonstrated by immunohistochemistry (IHC) for the proliferation marker Ki-67 (Fig. 1A), suggesting that FHL2 is dispensable for proliferation of intestinal epithelial cells. No significant difference was observed in the presence and localization of differentiated intestinal cell lineages (enterocytes, enteroendocrine, goblet cells and Paneth cells) in the FHL2 mutant mice (data not shown). We crossed the *Apc^Δ14/+^* strain on the C57Bl/6 background [32] into the *FHL2^−/−^* strain on the hybrid Black Swiss-129-SV/J background [33]. Intercrosses from the F1 generation produced *Apc^Δ14/+^* mice with three different *FHL2* genotypes. Only the F2 generation was used for further analysis in order to rule out any influence of genetic background. In contrast to *Apc^Δ14/+^* mice on the C57Bl/6 background which die at 6-month-old [32], Apc^Δ14/+^FHL2^+/+^ mice on the mixed genetic background do not succumb to intestinal adenomas until 20-month-old, which is a late stage in a mouse life (the life span of wt animals on the same genetic background was about 24-month-old). Because most of *Apc^Δ14/+^FHL2^−/−^* mice died at 24-month-old, the impact of *FHL2* deficiency on time course of death was not significant in *Apc^Δ14/+^* animals of this study (data not shown).

**Figure 2:**
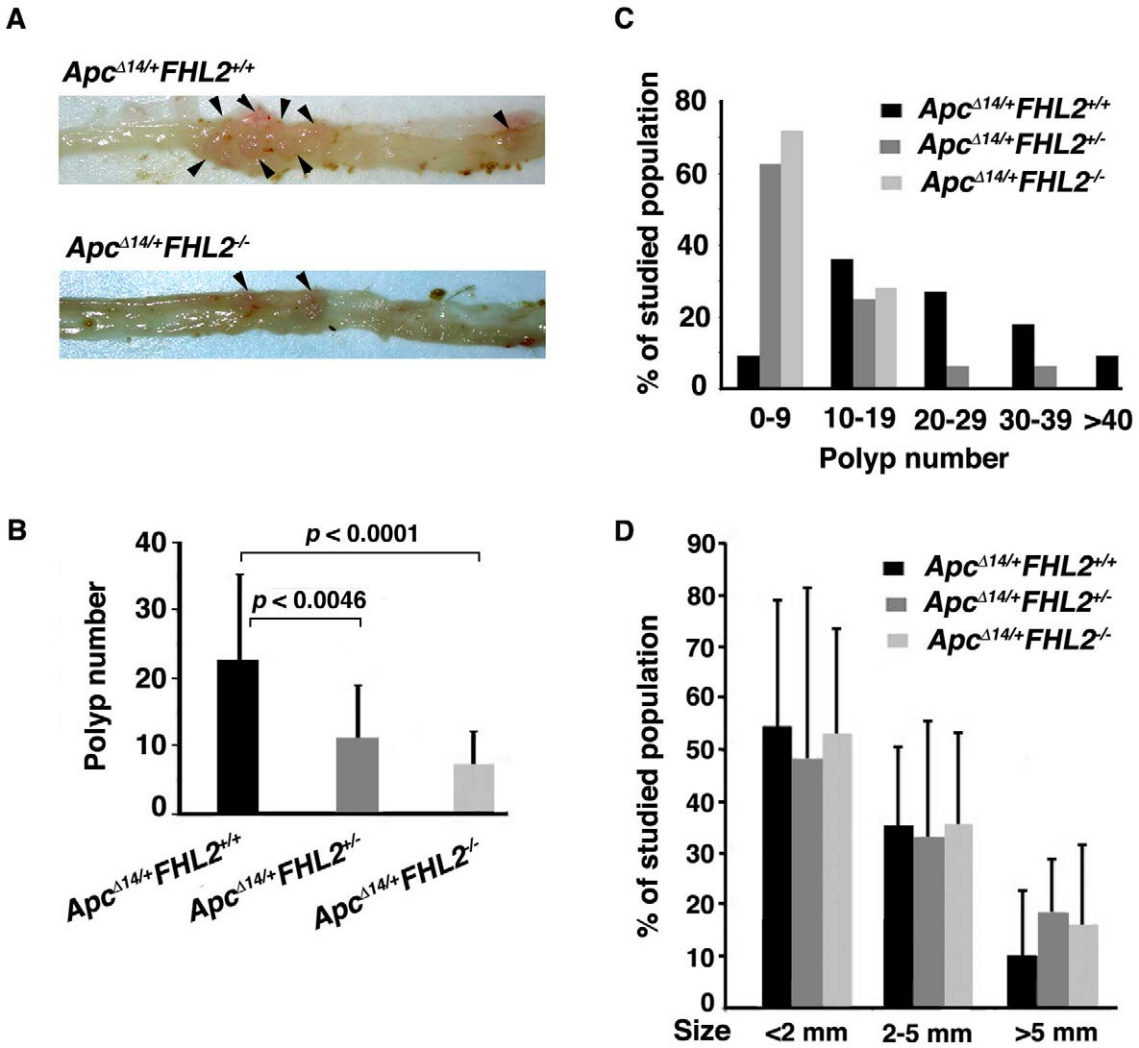
Loss of FHL2 reduces intestinal polyp multiplicity. (A) Representative images of intestines from 11-month-old *Apc^Δ14/+^FHL2^+/+^* and *Apc^Δ14/+^FHL2^−/−^* mouse. Note the marked decrease in the number of polyps in *Apc^Δ14/+^FHL2^−/−^* mice. (B) Total number of intestinal polyps was counted in 11 *Apc^Δ14/+^FHL2^+/+^*, 16 *Apc^Δ14/+^FHL2^−/+^* and 18 *Apc^Δ14/+^FHL2^−/−^* mice at 11-month-old. Compared to *Apc^Δ14/+^FHL2^+/+^* littermates, significant reduction in polyp number was observed in *Apc^Δ14/+^FHL2^−/+^* (*p*<0.0046) and *Apc^Δ14/+^FHL2^−/−^* mice (*p*<0.0001). (C) Polyp distributions are expressed as the percentages of mice having 0–9, 10–19, 20–29, 30–39 or more than 40 polyps in the intestine. (D) FHL2 deficiency had no effect on tumor growth. The percentages of polyps in each mouse with sizes less than 2 mm, between 2 to 5 mm and more than 5 mm are presented.

Mice were first sacrificed at 3 months. Analysis of the intestine revealed similar lengths for *Apc^Δ14/+^* mice of each FHL2 genotype (data not shown). We divided the intestinal tract into small intestine and colon segments and scored for polyp number, location, and size. Despite few adenomas developed at this stage, tumors occurred more frequently in *Apc^Δ14/+^FHL2^+/+^* animals (73%, n = 11) than in *Apc^Δ14/+^FHL2^−/−^* littermates (40%, n = 10). We then examined the intestine at 11 months. Compared to *Apc^Δ14/+^FHL2^+/+^* littermates, the polyp number in the small intestine decreased by 68% in *Apc^Δ14/+^FHL2^−/−^* mice and 52% in *Apc^Δ14/+^FHL2^+/−^* mice (Figs. 2A and 2B). Precisely, while the polyp number in *Apc^Δ14/+^FHL2^+/+^* could reach more than 40, the great majority of *Apc^Δ14/+^FHL2^−/−^* mice developed less than 9 tumors and none of the animals had more than 19 polyps (Fig. 2C). Analysis by the chi-square test showed that the difference in the sample distribution between *Apc^Δ14/+^FHL2^+/+^* and *Apc^Δ14/+^FHL2^−/−^* mice in Fig. 2C was statistically significant (*p*<0.0036), indicating that FHL2 deficiency inhibits polyp formation. To test the effect of FHL2 deficiency on tumor growth, we measured the sizes of polyps in *Apc^Δ14/+^FHL2^+/+^* and *Apc^Δ14/+^FHL2^−/−^* mice (Fig. 2D) and carried out Wilcoxon Rank Sum test to determine if there was a difference in tumor size between the two genotypes. The results showed that tumor sizes in *Apc^Δ14/+^FHL2^−/−^* mice were not significantly different from those in *Apc^Δ14/+^FHL2^+/+^* mice (*p*>0.2, Wilcoxon Rank Sum test), suggesting that loss of FHL2 activity does not affect tumor growth. Histological evaluation of the adenomas revealed no difference in gross histological characteristics or grade between *Apc^Δ14/+^FHL2^+/+^* and *Apc^Δ14/+^FHL2^−/−^* mice (see Fig. 1B). No difference was observed in the distribution of polyps throughout the intestine between *Apc^Δ14/+^FHL2^+/+^* and *Apc^Δ14/+^FHL2^−/−^* mice (data not shown). We next analyzed the lesions in the colon. Few polyps developed in the colon at 11 months in *Apc^Δ14/+^* mice with any of the *FHL2* genotypes. However, while tumors were detected in 45% of *Apc^Δ14/+^FHL2^+/+^* (n = 11), only 17% of *Apc^Δ14/+^FHL2^−/−^* animals (n = 18) developed polyps, indicating that the frequency of tumors in the colon is also decreased by FHL2 deficiency. We therefore conclude that loss of FHL2 suppresses tumor formation in the intestine by probably acting on the initiation of tumors.

### β-catenin and its targets cyclin D1 and c-myc are activated in adenomas independent of FHL2 genotype

**Figure 3:**
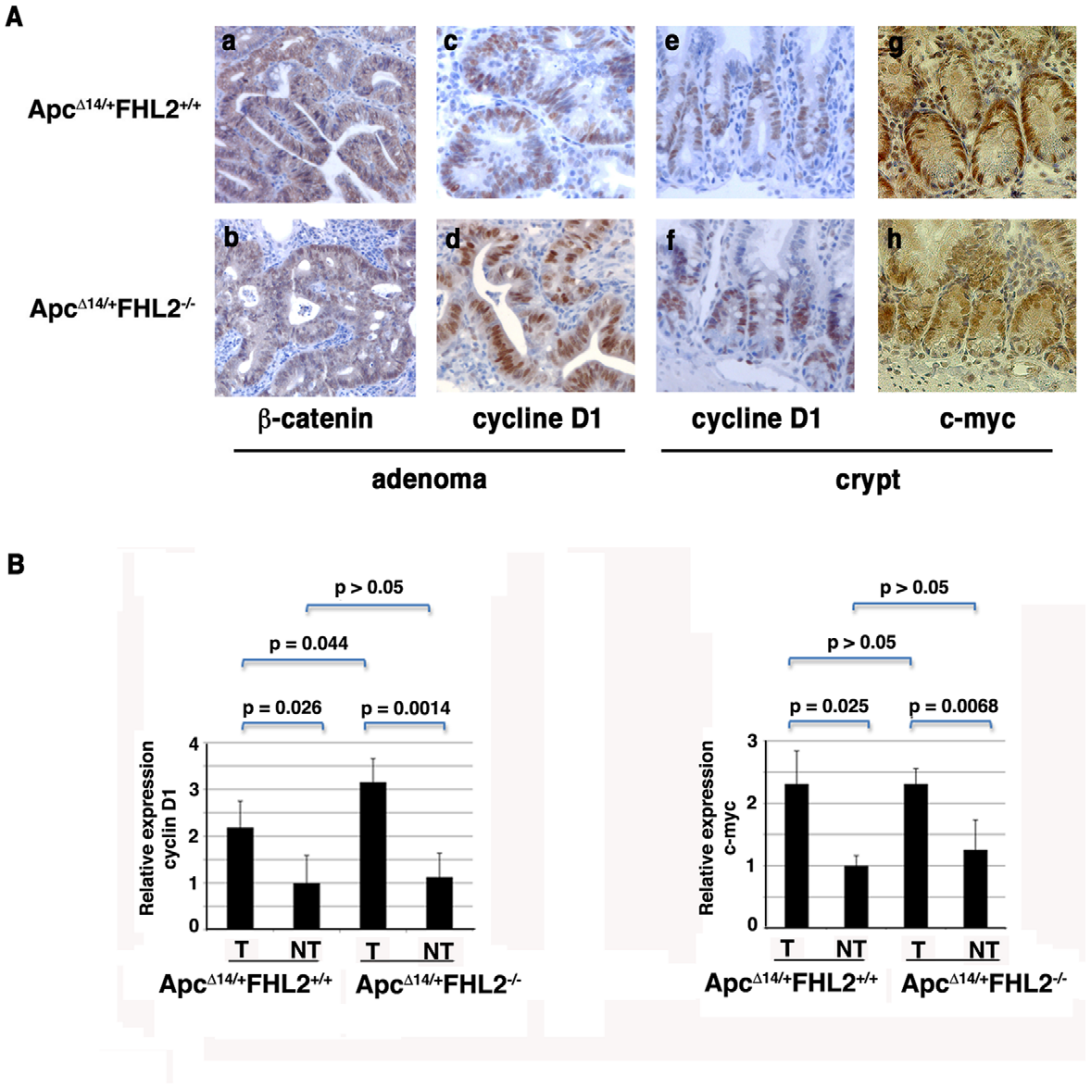
Activation of the Wnt signalling in the intestine of *Apc^Δ14/+^FHL2^−/−^*mice. (A) Immunostaining for β-catenin in adenomas (a, b), for cyclin D1 in adenomas (c, d) and normal intestine (e, f) and for c-myc in normal intestine (g, h) in *Apc^Δ14/+^FHL2^+/+^* (a, c, e, g) and *Apc^Δ14/+^FHL2^−/−^* mice (b, d, f, h). Sections were photographed at the same magnification. Original magnifications: X200. (B) Activation of the Wnt target genes cyclin D1 and c-myc in adenomas is unaffected by FHL2 deletion. Real-time RT-PCR analysis of cyclin D1 and c-myc expression was performed with intestinal tumor (T) and adjacent normal tissue (NT). Cyclin D1 and c-myc expression was normalized to 18S RNA and normalized values in normal intestine of *Apc^Δ14/+^FHL2^+/+^* was arbitrarily set at 1. The average values and standard deviations from two independent experiments from four mice of each genotype are shown. The p value is calculated according to Student's test.

We have previously shown that FHL2 interacts with β-catenin and is a crucial regulator of cyclin D1 expression in fibroblasts [5]. To investigate the molecular mechanisms of polyp formation in *Apc^Δ14/+^FHL2^−/−^* mice, we analyzed expression of β-catenin and its targets cyclin D1 and c-myc in tumors. Immunohistochemical staining revealed strong nuclear expression of β-catenin in polyps from both *Apc^Δ14/+^FHL2^+/+^* and *Apc^Δ14/+^FHL2^−/−^* mice (Fig. 3A, a and b), indicative of activation of the β-catenin/Wnt pathway. As direct targets of β-catenin, cyclin D1 and c-myc are closely involved in intestinal tumorigenesis associated with activation of Wnt signalling [35]–[38]. To assess the impact of FHL2 deficiency on the targets, we examined expression of cyclin D1 and c-myc at both RNA and protein levels in the intestine of *Apc^Δ14/+^FHL2^−/−^* mice. As shown in Fig. 3B, the mRNA levels of cyclin D1 and c-myc were indistinguishable in nontumor intestine between *Apc^Δ14/+^FHL2^+/+^* and *Apc^Δ14/+^FHL2^−/−^* animals, and were strongly increased in adenomas from mice of both genotypes. Whereas expression of cyclin D1 in adenomas from *Apc^Δ14/+^FHL2^−/−^* mice showed significant higher level than that in tumors from *Apc^Δ14/+^FHL2^+/+^* mice, no significant difference of c-myc expression was observed in adenomas between *Apc^Δ14/+^FHL2^+/+^* and *Apc^Δ14/+^FHL2^−/−^* animals (Fig. 3B). In parallel, immunohistochemical analysis showed nuclear expression of cyclin D1 and c-myc in the proliferative compartment of normal crypts in both *Apc^Δ14/+^FHL2^−/−^* and *Apc^Δ14/+^FHL2^+/+^* animals (Fig. 3A, e, f, g, and h). Moreover, in keeping with the proliferative property of tumor cells, cyclin D1 was highly expressed in all adenomas, regardless of the FHL2 genotype (Fig. 3A, c and d). In addition, we compared expression of other β-catenin targets such as Axin 2 and metalloproteinase matrilysin (MMP-7) between tumors and adjacent nontumorous tissues by real time RT-PCR [39], [40]. Axin 2 and MMP-7 were uniformly activated in tumor samples, with no significant difference among *Apc^Δ14/+^FHL2^−/−^* and *Apc^Δ14/+^FHL2^+/+^* animals (data not shown). We also performed IHC with the intestine of *Apc^Δ14/+^FHL2^−/−^* mice for c-Jun and peroxisome proliferators-activated receptor δ (PPARδ), which have been shown to be the Wnt targets critical for the control of intestinal tumorigenesis [41], [42]. The expression of c-Jun and PPARδ was similar between *Apc^Δ14/+^FHL2^−/−^* and *Apc^Δ14/+^FHL2^+/+^* mice in both normal and adenomatous tissues (data not shown). Taken together, these findings indicate that Wnt signalling remains constitutively active in the lesions of *Apc^Δ14/+^FHL2^−/−^* mice and that the inhibitive effects of FHL2 deficiency on intestinal neoplasia are not associated with defects in the activation of c-myc and cyclin D1.

### Loss of FHL2 increases cell migration

**Figure 4:**
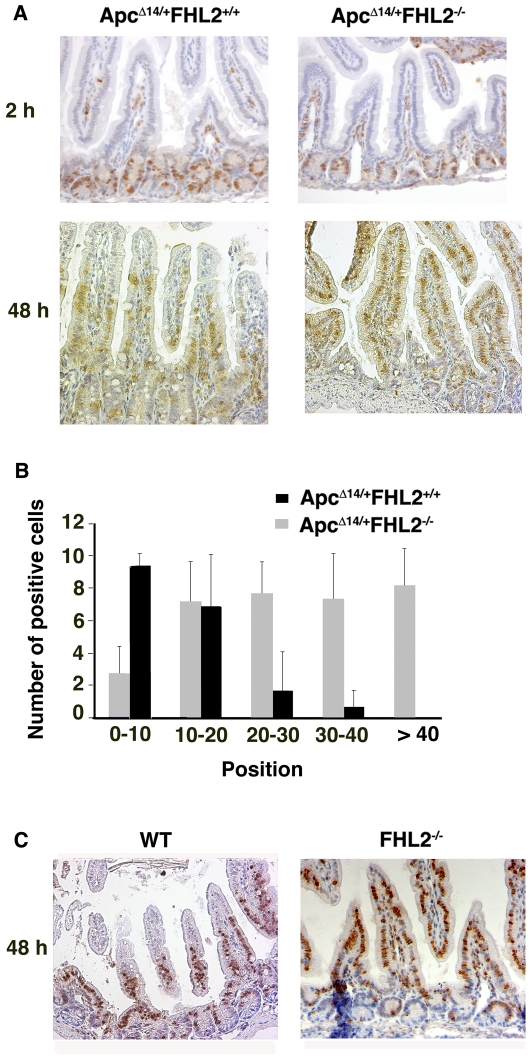
FHL2 deficiency accelerates enterocyte movement. (A) Representative images of BrdU-positive cells along the crypt-villus axis at 2 h and 48 h post-BrdU labelling in *Apc^Δ14/+^FHL2^+/+^* and *Apc^Δ14/+^FHL2^−/−^* mice. (B) Distribution of BrdU-positive cells at 48 h. The crypt base was set as position 0. Three mice of each genotype were analyzed. (C) Representative images of BrdU-positive cells along the crypt-villus axis at 48 h post-BrdU labelling in wt and *FHL2^−/−^* mice. Original magnifications: X200 (A and C).

Cell migration represents a fundamental mechanism involved in the maintenance of epithelial homeostasis in the intestine. Impaired cell migration correlates with tumor growth and neoplastic progression during development of colorectal cancer [43]. The role of FHL2 in assembly of extracellular matrix prompted us to examine the impact of FHL2 deficiency upon cell migration along the crypt-villus axis. *Apc^Δ14/+^FHL2^−/−^* and *Apc^Δ14/+^FHL2^+/+^* mice were administrated with BrdU by intraperitoneal injection and sacrificed 2 h and 48 h later for BrdU-positive cell scoring in longitudinal sections with the base of crypt set as position 0. As shown in Fig. 4A, no difference was observed between *Apc^Δ14/+^FHL2^−/−^* and *Apc^Δ14/+^FHL2^+/+^* in the position and the number of BrdU-positive cells after 2 h exposure to BrdU. At 48 h, however, *Apc^Δ14/+^FHL2^−/−^* cells moved to a higher position than *Apc^Δ14/+^FHL2^+/+^* cells along the crypt-villus axis (Fig. 4A). Indeed, an average of 87.4% labelled *Apc^Δ14/+^FHL2^+/+^* cells were located at positions between 0 and 20 in longitudinal sections versus 29.8% of labelled *Apc^Δ14/+^FHL2^−/−^* cells (*p*<6.10^−6^, Student's *t* test), and nearly 70% of BrdU-positive cells in *Apc^Δ14/+^FHL2^−/−^* mice already migrated to positions higher than 20 against 12.6% in *Apc^Δ14/+^FHL2^+/+^* animals (*p*<10^−6^), showing an accelerated migration rate (Figs. 4A and 4B). Moreover, the number of BrdU-positive cells in *Apc^Δ14/+^FHL2^−/−^* mice was significantly increased compared to *Apc^Δ14/+^FHL2^+/+^* littermates (*p*<0.005) (Figs. 4A and 4B). As we found no anomaly in the organization of proliferative and differentiated compartments in *Apc^Δ14/+^FHL2^−/−^* intestine (see Fig. 1A and data not shown), the increased number of BrdU-labelled cells in these animals might be attributed to the rapid movement of the enterocytes from the proliferative compartment to the top of villi. The impact of FHL2 on cell migration was independent of Apc mutations, as the intestinal cells in *FHL2^−/−^* mice moved also faster than wt cells along the crypt-villus axis at 48 h post-BrdU injection (Fig. 4C). These data indicate that loss of FHL2 activity has positive effects on cell migration. As accelerated cell migration by chemopreventive agents has been reported to be beneficial for eliminating deleterious cells, the tumor suppression function of FHL2 deficiency may be achieved in part through acceleration of cell migration in *Apc^Δ14/+^* mice.

### Up-regulation of FHL2 in human intestinal adenoma and carcinoma

**Figure 5:**
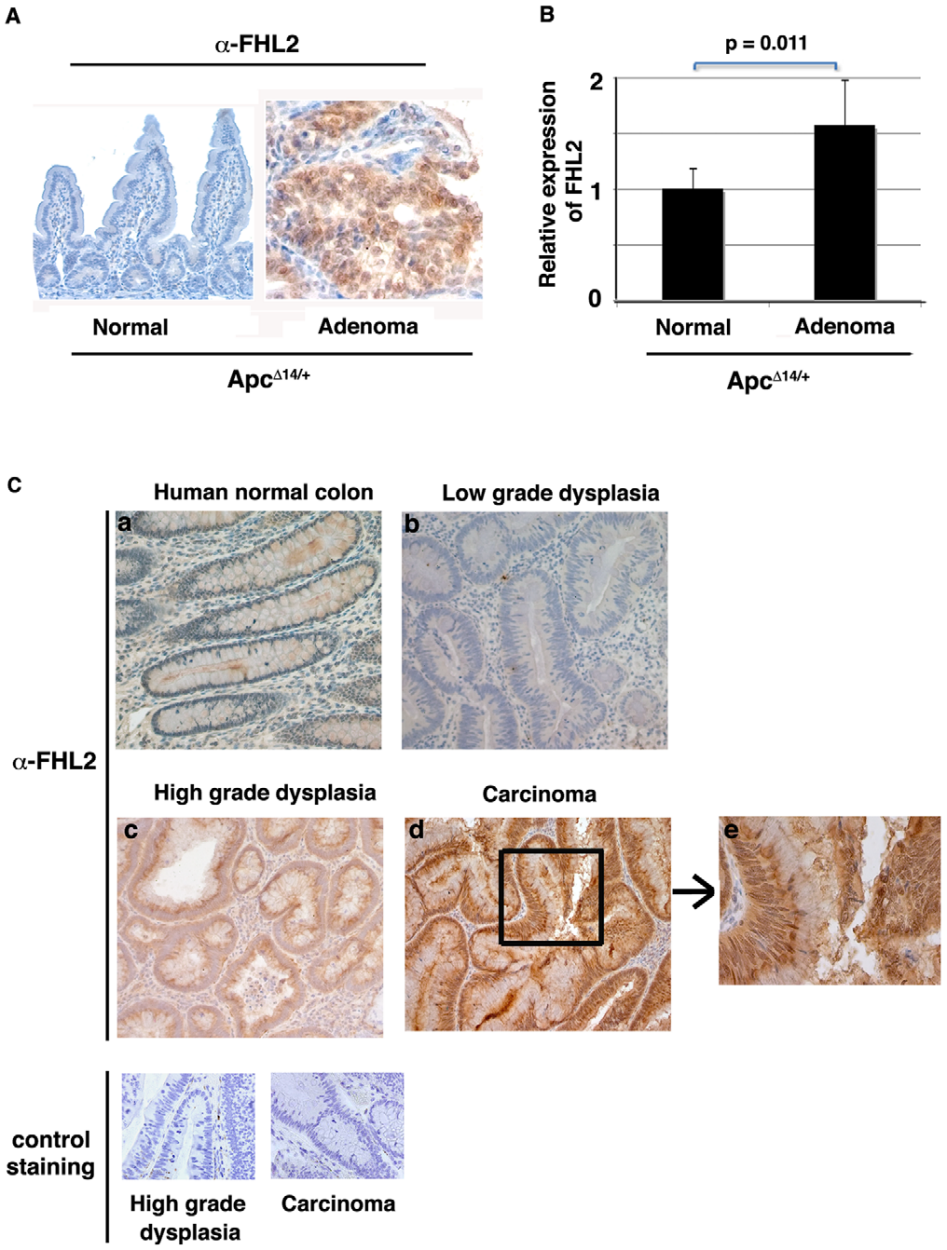
Up-regulation of FHL2 in intestinal tumors from *Apc^Δ14/+^* mice and human colon tumors. (A) FHL2 immunostaining of normal intestine and adenoma from *Apc^Δ14/+^* mouse. Original magnifications, X200. (B) Quantitative RT-PCR analysis of FHL2 expression in adenoma and adjacent normal tissue from five *Apc^Δ14/+^* mice. FHL2 expression was normalized to 18S RNA. The ratio of the FHL2/18S signal in NT was arbitrarily set at 1. The average values and standard deviations for three independent experiments from five *Apc^Δ14/+^* mice are shown. Average fold induction in adenomas is 1.73 (*p* = 0.011, Student's *t* test). (C) FHL2 expression in normal human colon (a), in human low-grade (b) and high-grade colon dysplasia (c) and in human colon carcinoma (d) by immunohistochemistry. Control staining on high-grade dysplasia and human colon carcinoma was performed as FHL2 staining without the primary anti-FHL2 antibody. Original magnifications, X200. Part of the image of human colon carcinoma was further enlarged to better visualize individual cells (e).

Next, we examined FHL2 expression in normal and tumorous tissues from *Apc^Δ14/+^* mice by IHC with anti-FHL2 antibody, which does not cross-react with any other protein, since it detects no signal in *Apc^Δ14/+^ FHL2^−/−^* intestine (data not shown). In contrast to normal epithelium where FHL2 signal was barely detectable, strong nuclear staining of FHL2 was observed in tumors developed in *Apc^Δ14/+^* mice (Fig. 5A). We then examined FHL2 expression in the intestine of *Apc^Δ14/+^* animals by real time RT-PCR. As shown in Fig. 5B, the level of FHL2 transcript was significantly increased in tumor samples, compared to normal adjacent intestinal tissues. To assess the expression of FHL2 in human tumors, we analyzed five human colon adenomas with low-grade dysplasia, five human colon adenomas with high-grade dysplasia and five carcinomas by IHC for FHL2. Staining of adjacent normal crypts was used as internal control. In keeping with a previous report [24], FHL2 protein, undetectable in normal tissues, was expressed in all high-grade dysplasia and carcinomas analyzed (Fig. 5C, compare a with c and d). Interestingly, barely detectable in low-grade dysplastic tissues, FHL2 was significantly increased in high-grade dysplasia and cancer cells (Fig. 5C, compare b with c and d), showing a progressive expression pattern. Moreover, contrasting markedly with the predominant cytoplasmic localization in high-grade dysplasia, intense nuclear accumulation of FHL2 protein was observed in carcinomas (Fig. 5C, e).

Taken together, these data suggest that FHL2 activation in intestinal cells may be associated with pre-cancerous and cancerous stages and that FHL2 protein may accumulate in the nucleus during neoplastic progression.

## Discussion

In this study, we provide new insights into the FHL2 function in intestinal tumorigenesis. By employing a genetic approach *in vivo*, we demonstrate that loss of FHL2 drastically suppresses tumorigenesis in the murine intestine, which may be partly due to the positive effects of FHL2 deficiency on cell migration in intestinal epithelium. In the study of human colorectal tumors, we found that FHL2 expression might be closely related to disease stages: the level of FHL2 in high-grade dysplasia is significantly elevated compared to normal tissues as well as low-grade dysplasia but clearly below that in carcinomas. Moreover, cellular localization of FHL2 switches from predominant cytoplasmic expression in high-grade dysplasia to enhanced nuclear accumulation in cancer cells.

The *Apc^Δ14/+^FHL2^−/−^* mouse model underscores that animals deficient for FHL2 are less susceptible to oncogene-induced tumors. However, how FHL2 is involved in the proliferative and transforming functions of the Wnt signalling in the intestine is not clear. Previous report has shown that the D-type cyclins, in particular cyclin D1, are dramatically down-regulated in *FHL2^−/−^* fibroblasts [5]. The results of this study indicate that cyclin D1 expression is not dependent of FHL2 in the context of intestinal epithelium. It appears that cyclin D1 expression was stronger in tumors developed in *Apc^Δ14/+^FHL2^−/−^* mice than those in *Apc^Δ14/+^FHL2^+/+^* animals. As FHL2 plays an important role in cell proliferation, it is possible that high level of cyclin D1 may counteract FHL2 deficiency in the stimulation of tumor cell growth in *Apc^Δ14/+^FHL2^−/−^* mice. We also observed that the expression of other β-catenin targets including c-myc, Axin2, MMP-7, c-Jun and PPARδis not dependent of FHL2 in the intestine. Further analysis of the involvement of Wnt and FHL2 signalling in this specific tissue should provide insight into whether the Wnt and FHL2 signalling pathways function in an interdependent regulatory network.

The loss of FHL2 activity in intestinal epithelium clearly accelerates cell migration, which constitutes a fundamental mechanism in the control of tissue homeostasis. Apc mutation in *Apc^min/+^* mice is associated with decreased cell migration, resulting in a prolonged residence time for enterocytes in the intestine [43]. Chemopreventive agents presumably exert their anti-tumor effects by increasing the rates of cell migration, thus eliminating rapidly deleterious cells [44]–[46]. The mechanism of the increase of cell migration in FHL2 mutant intestine remains to be elucidated. Because FHL2 is involved in focal adhesion, ECM-cell interaction and assembly of the extracellular matrix [4], [5], a role for FHL2 in the regulation of cell mobility seems an attractive possibility. The tumorigenic effects of proteins that regulate cell migration have been previously illustrated by SPARC, a matricellular protein that associates with the extracellular matrix and modulates cell-ECM interaction [47]. Deficiency of SPARC augments enterocyte movement in *Apc^min/+^* mice, thereby suppressing adenoma formation [47]. Moreover, the positive effects of FHL2 mutation on cell migration in *Apc^Δ14/+^FHL2^−/−^* mice correlate with the observation that FHL2 deficiency perturbs essentially adenoma initiation.

Consistent with an early report showing nuclear expression of FHL2 in gastrointestinal cancerous tissues [24], our data further indicate that high level expression and nuclear accumulation of FHL2 in human colorectal cancer might reflect disease progression towards the malignant state. This observation is correlated with recent discoveries that invasive breast cancers have much stronger expression of FHL2 than premalignant ductal carcinoma in situ samples [25] and that increases of nuclear FHL2 in prostate cancer are strongly correlated with dedifferentiation of cancer cells and with high Gleason grade [6]. Altogether, these results support the view that the intensity and localization of FHL2 expression in cancer cells may serve as a biomarker in classifying tumor stage and predicting disease outcome [27]. Indeed, breast cancer patients with tumors expressing low amounts of FHL2 have a better survival compared to those with high intra-tumoral FHL2 expression [28] and activation of nuclear FHL2 signalling is linked to aggressiveness and recurrence of prostate cancer [27]. In colorectal cancer, further examination of FHL2 expression in a large panel of samples may permit to determine if FHL2 could be used as a marker for stage classification of the disease. Mechanistically, the sensor function and transcription coregulator activity of FHL2 can provide a ready explanation for the up-regulation and nuclear translocation of FHL2 in highly malignant cells. In prostate cancer cells, FHL2 is strongly induced by androgens [48]. As a coactivator of AR, FHL2 in turn robustly stimulates the AR activity that is critical for prostate cancer progression [48], [49]. In colorectal cancer, the molecular hallmark is the accumulation of β-catenin in the nucleus. However, expression of the β-catenin target genes analyzed in this study seems not be affected by FHL2 deficiency. Nevertheless, up-regulation of FHL2 and its nuclear accumulation may stimulate transcription activity of not yet uncovered β-catenin targets or other transcription complexes, which may be required for carcinogenic progression.

Our data highlight FHL2 as important molecule in mediation of the transformation process and suggest that disruption of the FHL2 signalling may provide a viable and specific strategy for therapeutic intervention in colorectal cancer.

## Materials and Methods

### Mice

Male *Apc^Δ14/+^* animals on the C57BL/6 background were crossed with female *FHL2^−/−^* mice on the hybrid Black Swiss-129-SV/J background [32], [33]. F1 *Apc^Δ14/+^ FHL2^+/−^* mice were intercrossed. Only the F2 *Apc^Δ14/+^* mice with the three *FHL2* genotypes were included in the study. Mice were housed under pathogen-free conditions. All animal procedures were carried out in accordance with French government guidelines. All experiments involving mice have been approved by Institut Pasteur.

### Tumor scoring and histopathological analysis

Intestines were removed from F2 *Apc^Δ14/+^* mice with different *FHL2* genotypes at 3 or 11 months and fixed in 4% PFA. Polyps were counted and measured using a Nikon dissecting microscope at×6 magnification by an observer blinded to the genotype of the mice. Intestines were rolled in the “Swiss rolls” configuration and proceeded for paraffin embedding [32].

### Immunohistochemistry

Sections were cut from paraformaldehyde (PFA)-fixed paraffin-embedded ‘Swiss rolls’. Tissues were dewaxed in xylene and unmasked in a citric acid solution (H-3300, Vector Laboratories) at 96°C for 45 min. Normal horse serum (2.5%; S-2000, Vector Laboratories) was used as blocking solution. Sections were incubated at room temperature with primary antibodies for 60 min. Endogenous peroxidase activity was blocked by incubating sections with 3% hydrogen peroxide. The sections were then incubated with secondary antibodies (Vector Laboratories) for 30 min. The peroxidase reaction was developped using DAB Substrate Kit (SK-4100, Vector Laboratories). Hematoxylin was used for counterstaining. Antibodies against β-catenin and cyclin D1 (NeoMarkers), FHL2 (MBL), c-myc (Santa Cruz) and Ki-67 (Novocastra) were used as primary antibodies. Images were obtained on a FXA Microphot microscope equipped with a Nikon D1 camera controlled by Nikon capture.

### Quantitative RT-PCR analysis

Total RNA was extracted from polyps and adjacent non tumoral intestines removed from 11-month-old *Apc^Δ14/+^* mice. Real-time PCR was performed as described previously [5].

### Measurement of enterocyte migration

Mice at 10 weeks were administrated with Bromodeoxyuridine (BrdU) (50 mg/kg mouse body weight), followed by immunohistochemical analysis with anti-BrdU (Dako). Fields containing crypt transverse sections were selected randomly at several locations for BrdU positive cell counting in crypt-villus units.

### Human polyp samples

Fifteen human colon specimens were obtained in Hôpital Bégin at Saint Mandé and fixed in PFA. Informed consent of patients was obtained at the hospital and the study was performed in accordance with European Guidelines for biomedical research.
